# Supplementary material for: Identification of photoperiod-induced specific miRNAs in the adrenal glands of Sunite sheep (Ovis aries)
Source: Front Vet Sci. 2022 Jul 22;9:888207. doi: 10.3389/fvets.2022.888207 (PMC9354845; doi:10.3389/fvets.2022.888207)
Supplement: Supplementary file 1 [file Data_Sheet_1.ZIP › Supplementary file/miRDeep2 file.zip!/miRDeep2 file/Know_Structure/SPLP42A3/oar-mir-152.pdf]

miRBase precursor : oar-mir-152
Total read count : 10589
oar-miR-152 read count : 10589
remaining reads : 0

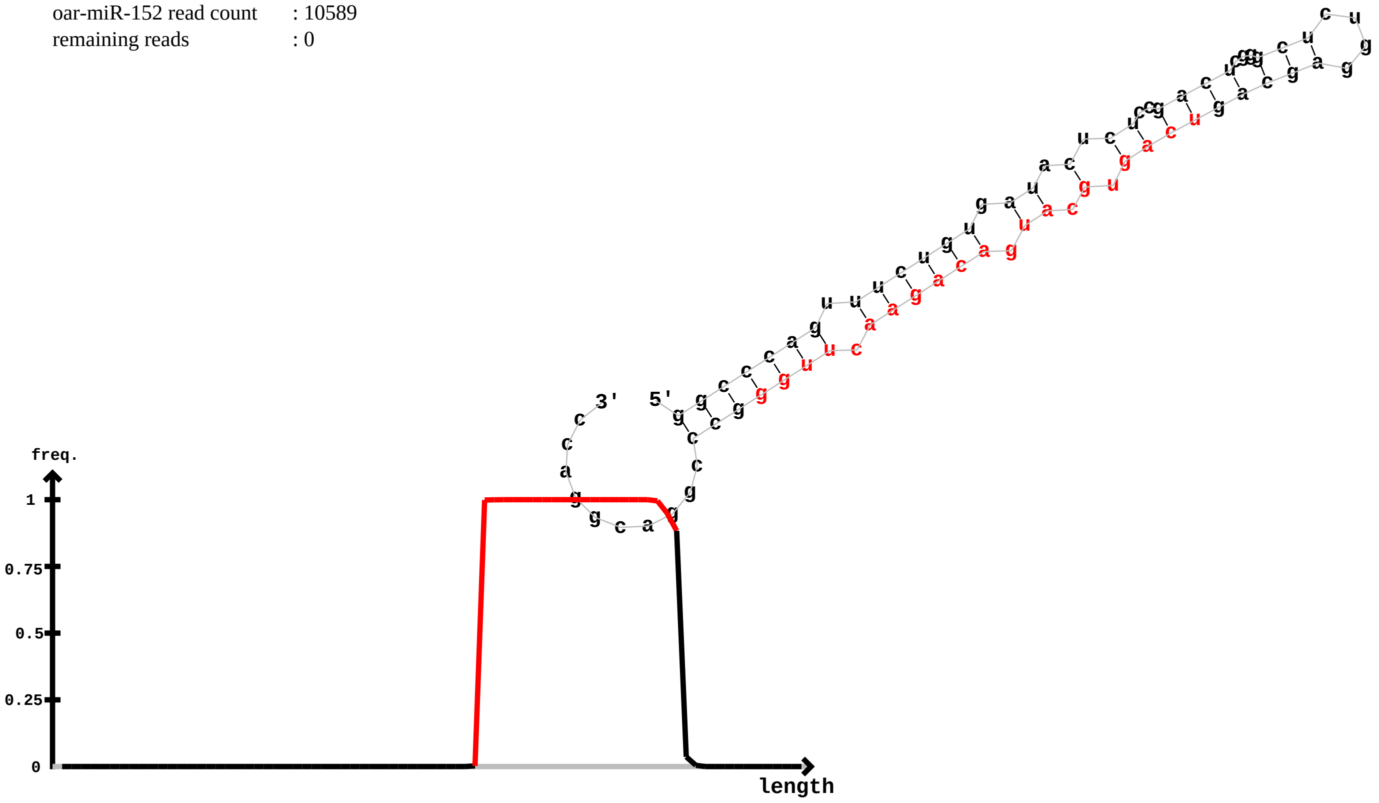


```
                                         oar-miR-152
5'- ggcccaguuucugugauacucuccgacucgggcucuggagcagucagugcaugacagaacuugggcccggacggacc   -3'    exp
    (((((((.((((((.((.(.((..((((...(((....))))))))).).)).)))))).)))))))..........     reads      mm        sample
    .........................................agucagugcaugacagaacuugg.............     1          0         seq
    ..........................................gucagugcaugacagaacuug..............     7          0         seq
    ..........................................gucagugcaugacagaacuugg.............     19         0         seq
    ...........................................ucagugcaugacagaac.................     1          0         seq
    ...........................................ucagugcauCacagaacu................     7          1         seq
    ...........................................ucagugcaugacagaacu................     41         0         seq
    ...........................................ucagugcauCacagaacuu...............     8          1         seq
    ...........................................ucagugcaugacagaacuu...............     472        0         seq
    ...........................................ucagugcaugacagaacuug..............     694        0         seq
    ...........................................ucagugAaugacagaacuug..............     2          1         seq
    ...........................................ucagugcaugacagaacuugg.............     8940       0         seq
    ...........................................ucagugcaugacagaacuuggg............     336        0         seq
    ...........................................ucagugcaugacagaacuugggc...........     49         0         seq
    ............................................cagugcaugacagaacuug..............     2          0         seq
    ............................................cagugcaugacagaacuugg.............     5          0         seq
    .............................................agugcaugacagaacuu...............     1          0         seq
    .............................................Cgugcaugacagaacuug..............     1          1         seq
    .............................................agugcaugacagaacuugg.............     3          0         seq
```
